# Allometry of Defense: Predator Shift Alters Ontogenetic Growth Patterns in an Antipredator Trait

**DOI:** 10.3390/insects14080712

**Published:** 2023-08-17

**Authors:** Bin Jiang, Yu Yao, Rüdiger Mauersberger, Dirk J. Mikolajewski

**Affiliations:** 1 Anhui Provincial Key Laboratory for Conservation and Utilization of Important Biological Resources, College of Life Sciences, Anhui Normal University, Wuhu 241000, China; yao_yu123@163.com; 2 Institut für Biologie, Freie Universität Berlin, 14195 Berlin, Germany; d.mikolajewski@fu-berlin.de; 3 Förderverein Feldberg-Uckermärkische Seenlandschaft e.V., 17268 Templin, Germany; mauersberger@uckermaerkische-seen.de

**Keywords:** predator–prey interactions, antipredator traits, allometry, spines, dragonfly larvae

## Abstract

**Simple Summary:**

Predators drive prey trait diversification and promote ecological speciation. The impacts of predation are not only on the final state of antipredation traits, but also on the development of antipredation traits. Species of the dragonfly genus *Leucorrhinia* are distributed in both habitats dominated by predatory fish (fish lakes) and habitats dominated by predatory invertebrates (invertebrate lakes). In larval dragonflies, the spine is one of the most efficient traits deterring gape-limited fish predators. However, the spine is not useful in invertebrate lakes. In this study, we compared the developmental patterns of spines in both habitats. We constructed the scaling relationship between spine length and body size and compared the inflexion point on those curves in five species of *Leucorrhinia* dragonfly larvae. Here, we found that fish-lake *Leucorrhinia* species kept a higher spine growth rate than species from invertebrate lakes, and *Leucorrhinia* species from fish lakes displayed accelerated spine growth rate at larger body size compared to invertebrate-lake species. Our results highlight that development patterns, as well as the final states of antipredator traits, are essential to understanding predator–prey interactions.

**Abstract:**

Predation is a major factor driving prey trait diversification and promoting ecological speciation. Consequently, antipredator traits are widely studied among prey species. However, comparative studies that examine how different predators shape the ontogenetic growth of antipredator traits are scarce. In larval dragonflies, abdominal spines are effective traits against predatory fish in fish lakes, which prefer larger prey. However, defensive spines increase mortality in habitats dominated by invertebrate predators (invertebrate lakes), which prefer smaller prey. Thus, species from fish lakes may accelerate spine growth at a later body size compared to species from invertebrate lakes when growing into the preferred prey size range of predatory fish. In this study, we constructed the allometric relationship between spine length and body size and compared the inflexion point of those growth curves in five species of *Leucorrhinia* dragonfly larvae. We found that fish-lake *Leucorrhinia* species accelerated spine growth at a larger body size than congenerics from invertebrate lakes. Further, rather than extending spine length constantly through development, fish-lake species rapidly accelerated spine growth at a larger body size. This is likely to be adaptive for avoiding invertebrate predation at an early life stage, which are also present in fish lakes, though in smaller numbers. Our results highlight that comparative studies of ontogenetic patterns in antipredator traits might be essential to develop an integrated understanding of predator–prey interactions.

## 1. Introduction

Predators do not just affect population and species abundances [1], but are well-known major selective drivers mediating species trait diversifications [2]. Thus, diversification of antipredator traits has been intensively studied in morphology, behavior, physiology, and life history [3,4,5,6,7]. However, less well-understood are ontogenetic growth trajectories of antipredator traits [8,9,10]. Understanding patterns of allometric growth in antipredator traits (i.e., allometry of defense) is especially important for understanding the diversity of vital morphological traits for survival [11,12], and thus permits a more specific understanding of alterations in the timing and rate of ontogenetic events of defensive traits [13,14].

Many aquatic animals separate along the predator-mediated habitat gradient [15] with permanent waters either dominated by predatory fish or invertebrate predators. Thereby, both predator types differ in a number of foraging and hunting attributes, leading to habitat-specific morphological, behavioral, physiological, and life history adaptations in a number of groups (e.g., amphibian larvae [16], mayflies [17], etc.). Evolutionary changes in morphological antipredator traits might occur either by modifying size or shifting proportions among different body parts during development [18]. For example, *Leucorrhinia* species that have adapted to predatory fish have developed wider abdomens than species that have adapted to invertebrate predators, given that swim escape speed to evade predatory fish is linked to increased muscle content [19]. Further, small turtles grow wider faster than they grow longer, which enables them to quickly achieve size refuge from gape-limited predators (e.g., dolphinfish) [20]. *Diplodus* fish species change growth trajectories of body shape (from rapid changes to slow changes) when they reach a size range at which a habitat transition (from pelagic to necto-benthic habitat) occurs [21]. Allometric growth of defensive organs will evidently alter the defensive ability and influence the defensive strategies in animals [22].

Defensive spines are prominent features across the animal kingdom [17,23,24,25,26]. Abdominal spines acting as defensive traits are particularly well-studied among dragonfly larvae [27,28,29,30,31]. However, species differ in the occurrence of abdominal spines. Ancestrally occurring in fish lakes, *Leucorrhinia* species reduced the length of their spines after inhabiting invertebrate lakes [32,33]. Among species of the dragonfly genus *Leucorrhinia*, species preferring habitats dominated by predatory fish (hereafter fish-lake species) possess long defensive abdominal spines. This is due to an increased survival with long spines [34]. However, spines in *Leucorrhinia* species preferring habitats dominated by invertebrate top-predators (hereafter invertebrate-lake species) became either lost or reduced, contrasting to the species from lakes with predatory fish [32]. This is due to an increased mortality of larvae with long spines in the presence of large invertebrate predators [35].

As spine expressions differ distinctly between fish-lake species and invertebrate-lake species, ontogenetic growth of spines might show different trajectories. Invertebrate predators prefer smaller prey than predatory fish [36]. Thus, prey in invertebrate-lake species could decelerate growth of spines at an early developmental size because of spine-mediated mortality (see above), saving production costs of spines [37]. In contrast, predatory fish prefer larger prey, with prey often growing into the preferred size range of predatory fish during later development stages [38]. Long spines enlarge the body volume of dragonfly larvae, resulting in decreased mortality [28,34,39]. However, even though fish lakes are dominated by predatory fish, they also have invertebrate predators, though at low density [40]. Thus, when dragonfly larvae are too tiny to be detected by predatory fish but face the pressure of invertebrate predators in fish lakes, they might keep a low growth rate of the spines at early age; in contrastive, when they grow larger and are easily detected by fish, they might grow defensive spines rapidly.

Although the importance of defensive spines as an effective antipredator trait is known, evolutionary change of ontogenetic patterns of spine growth have not been examined in detail. Here, we investigated the evolutionary divergence on ontogenetic growth trajectories of lateral spine length over body size in five larval European *Leucorrhinia* species. Specifically, we hypothesized that larvae of fish-lake *Leucorrhinia* species should express defensive spines in a later developmental stage and have a larger growth rate of defensive spines than invertebrate-lake species.

## 2. Materials and Methods

### 2.1. Collecting and Larvae Breeding

**Figure 1:**
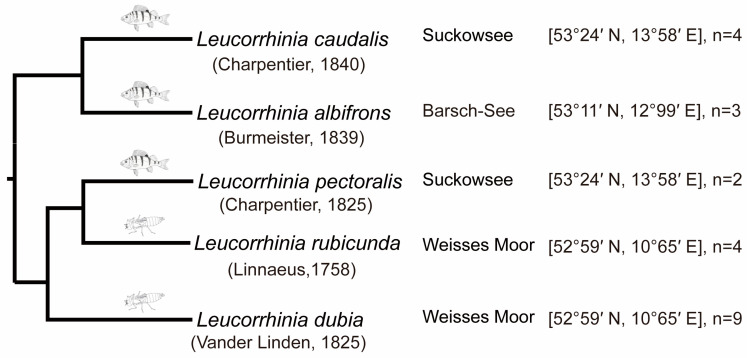
Phylogeny of five European *Leucorrhinia* species modified from Hovmöller and Johansson [32]. Fish and dragonfly larvae symbols indicate the preferred top predator (fish predator and large invertebrate predator, respectively) for each species. Sample locations and the number of egg clutches collected are given for each species.

At least two egg clutches for each *Leucorrhinia* species were collected during June 2016 (details in Figure 1). The common predatory fish species for *Leucorrhinia* in the sampling area include perch, crucian carp, pike, common roach, bream, etc. The common large invertebrate predators include *Anax, Aeshna* larvae, etc. Egg clutches were kept separately in 500 mL containers filled with 400 mL dechlorinated tap water until they hatched (egg hatching takes around 2 weeks in all species). From the end of June until the middle of July 2016, hatched larvae from the different clutches of same species were mixed and put into 10 L big buckets filled with 9 L dechlorinated tap water. Larvae were fed with *Daphnia* sp. *ad libitum* every second day. All buckets were kept outside on the campus of the Freie Universität Berlin [52°31′ N and 13°24′ E].

### 2.2. Growth Inspection and Measurements

Larvae were measured from November 2016 to September 2017 (December 2016 to March 2017 were excluded because they barely grow during this cold period). For each time point, we counted the number of larvae. Head and lateral spines on segment 8 and 9 of the abdomen were photographed for each larvae using an Olympus digital microscope SZX16 (Hamburg, Germany). We could only measure lateral spines by photographing larvae from above, because early larvae do not survive when handled with forceps to put them into the correct position. We concentrated on lateral spines and excluded dorsal abdominal spines because larvae do not cope well with the necessary handling for photographing the dorsal site at an early stage. We used photos to measure head width and lateral spines in segments 8 and 9 with the free software ImageJ 1.50 g (National Institutes of Health, Bethesda, MD, USA 2016). Head width was used as a surrogate for body size [41].

### 2.3. Data Analysis

All the analyses were carried out under R 4.3.0 [42]. Head width differences as well as lateral spine lengths of abdominal segments 8 and 9 in last instar larvae were analyzed using separate phylogenetic generalized least squares (PGLS) in the “caper” package [43] with predator regime (fish lake vs. invertebrate lake) as an independent variable and the average of each measurement in each species as a dependent variable (the significance level was 0.05). We incorporated branch length from a pruned *Leucorrhinia* phylogeny (Hovmöller pers. communication, Figure 1) [32].

Allometric relationships of lateral spines (length of spines in segments 8 and 9 measured in each time point of each larva, separately) over head width were fitted with different growth models (linear models and non-linear models, model description in supplementary material Table S1 following Paine et al. [44]). New sets of nonlinear allometries, such as logistic or Gompertz growth models, can be used to analyze the changes of the growth curves during the evolution of the phenotype [18,44]. Inflexion point indicates the point with the highest growth rate, in which the second derivative becomes “zero”. Before the inflexion point, the trait has a fast growth rate; after the inflexion point, the growth rate decelerates [13].

The most suitable models were selected according to AIC values. The head width values on the inflexion points of the curves for each spine (hereafter inflexion point) and the maximum slope for the growth of spines (hereafter maximum slope) were extracted from the best fitting models.

We tested whether predator regime (fish lake vs. invertebrate lake) affected the inflexion point and maximum slope using PGLS with predator regime as an independent variable (see the above PGLS analyses).

## 3. Results

**Figure 2:**
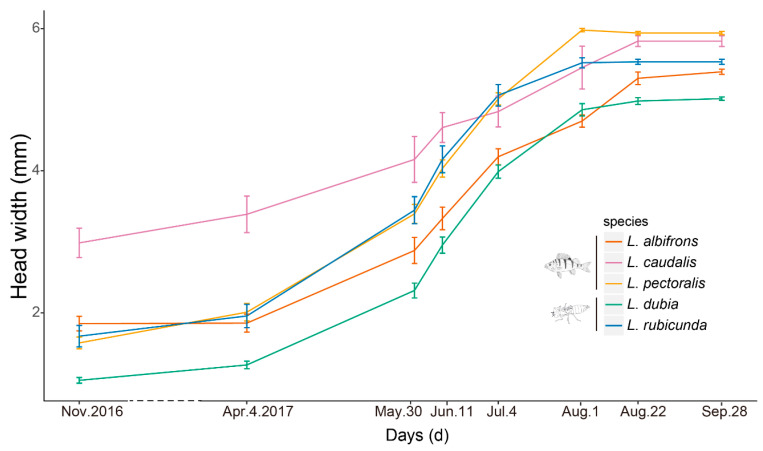
Examined time points and growth lines of each species. Presented are mean ± 1.95 SE (95% confidence intervals). Species and the top predator in their preferred habitat (fish predator and large invertebrate predator, respectively) are also indicated with fish and dragonfly larvae symbols. The dash line indicates the long time intervals during winter.

**Figure 3:**
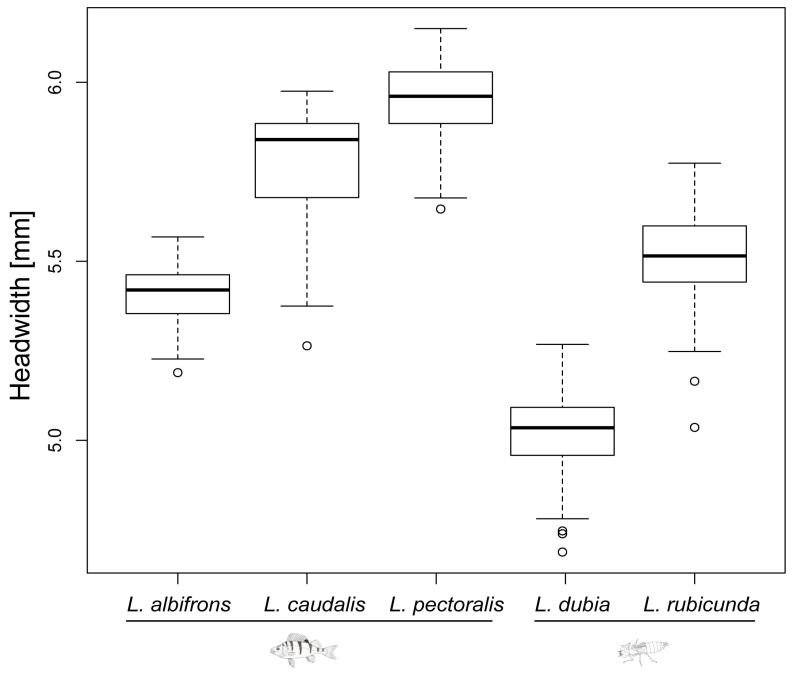
Head width of last instar larvae for all 5 *Leucorrhinia* species. Fish and dragonfly larvae symbols indicate the preferred top predator (fish predator and large invertebrate predator, respectively) for each species.

**Figure 4:**
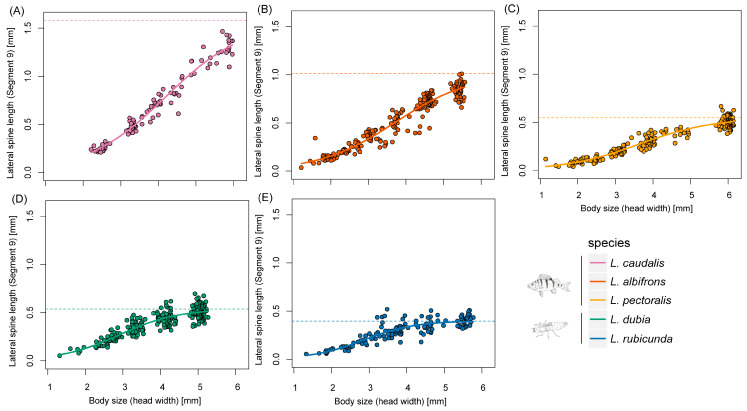
Scaling relationships of lateral spine length in segment 9 and body size during larval developing time. (**A**–**E**) represent different *Leucorrhinia* species, which are shown with different colors.

**Figure 5:**
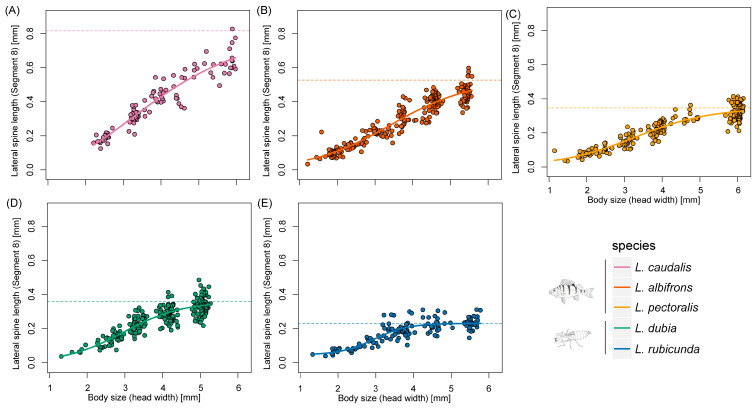
Scaling relationships of lateral spine length in segment 8 and body size during larval developing time. (**A**–**E**) represent different *Leucorrhinia* species, which are shown with different colors.

Last instar larvae of species from fish lakes were larger than from invertebrate lakes (F_1,3_ = 12.34, *p* = 0.039) (Figure 2 and Figure 3). Last instar lateral spines 9 (F_1,3_ = 6.77, *p* = 0.080) and spines 8 (F_1,3_ = 8.66, *p* = 0.060) only showed a tendency for being longer in fish lakes than invertebrate lakes (Figure 4 and Figure 5).

According to AIC, the three-parameter logistic model was the most suitable model for the allometric relationship between spines at segment 9 and head width across all species (Table 1). For spines at segment 8, four-parameter logistic model was the most suitable model in *L. caudalis* and the Gompertz model was best in *L. rubicunda*, while the other species were all fit with the three-parameter logistic model (Table 1 and Table S2).

**Figure 6:**
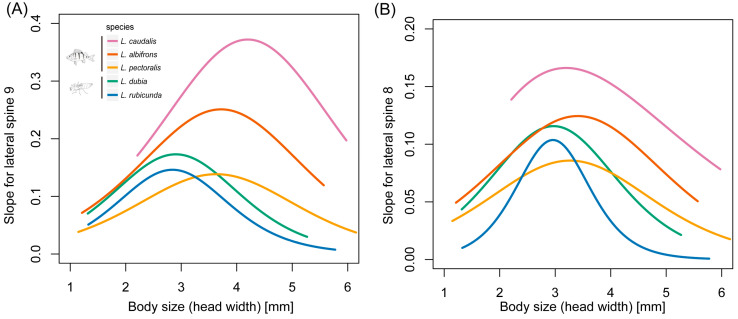
Slopes of the scaling relationship curves of lateral spine length and body size during larval developing time in 5 *Leucorrhinia* species. (**A**) Lateral spines in segment 9 and (**B**) lateral spines in segment 8.

Comparative analyses with PGLS showed that the inflexion point was significantly larger in fish-lake species than invertebrate-lake species in lateral spines at segment 9 (F_1,3_ = 23.19, *p* = 0.017) and 8 (F_1,3_ = 278.63, *p* < 0.001) (Figure 6, Table S2). The maximum slope for spines at segment 9 (F_1,3_ = 10.47, *p* = 0.048) and 8 (F_1,3_ = 10.58, *p* = 0.047) was also significantly larger in fish-lake species than invertebrate-lake species.

## 4. Discussion

Our study demonstrates that predator preferences caused evolutionarily shifts in ontogenetic growth trajectories of antipredator spines with growth of spines in fish-lake species accelerating at a larger body size and higher growth rate than in invertebrate-lake species. Thus, predators do not only select for more or less pronounced antipredator features, but also for a fine-scaled ontogenetic framework of antipredator traits supporting efficient defenses.

Predatory fish are gape-limited predators [45]. Prey that have evolved with large body dimensions can always survive better [46]. Preys enlarge their body dimensions in two ways: either enlarging their body size or growing longer spines. Our results show that fish-lake species evolve to have a larger body size than invertebrate-species, which indicate that large body size might be the important trait against gape-limited fish predators in *Leucorrhinia* species. The existence of spines could help to enlarge the body dimensions and increase predator handling time [39,47]. In this study, larvae of species from fish lakes exhibited only mildly longer lateral spines than larvae of species from invertebrate lakes. Longer spines in larval *Leucorrhinia* species from fish lakes have been shown several times [28,31,34]; these studies usually investigated the combined dorsal and lateral abdominal spines. However, in this study, we only measured lateral spines (see methods) and note that the change in dorsal and lateral spine length are positively correlated [33], and selection by predation on defensive spines is stronger in dorsal than in lateral spines [34], explaining why we only had a tendency for longer spines in fish-lake species.

Defensive traits are highly related and evolving as a whole [48]. Former experiments showed that in freshwater snails, behavioral and shape-based defenses were positively correlated [48]. Fish-lake species *L. caudalis* has the longest spine and a relatively large body size, which could be seen as trait cospecialization (Figure 3, Figure 4 and Figure 5). However, a compensatory relationship between spine length and body size was found in *L. pectoralis*. *L. pectoralis* possesses the shortest spine but the largest final body size among fish-lake species. Therefore, even under the same selection pressure from gape-limited fish predators, species can acquire different tactics to achieve large body dimensions.

Fish-lake species accelerated larval spine growth at a larger body size than invertebrate-lake species. This is likely to be evolutionary adaptation. Enlarging body dimension becomes an effective trait in defending against those gape-limited predators [39,49]. Thus, when fish-lake larvae grow into the size-relevant body size for predatory fish, elongated spines are an efficient way to avoid predation by increasing body volume [34]. Further, elongated larval spines in fish-lake species come with a larger final body size in fish-lake species (see results), which in combination increases body volume even further. However, spine length growth was not employed from an early developmental stage, but increased rather rapidly later in life. This might be due to the fact that predatory fish-dominated lakes also keep a low number of invertebrate predators [15,40]. Invertebrate predators prefer smaller prey size than predatory fish [36] and spines facilitate prey capture [35]. Consequently, larval *Leucorrhinia* in fish lakes should avoid development of long spines at an early stage, when they are not yet in focus by predatory fish, to prevent increased predation by invertebrate predators. Thus, growth differentiation of defensive spine seems strictly synchronized with potential costs and benefits of the defense at different life stages [10].

Spine trait divergence due to predators could stem either from a long evolutionary adaptation or simply from phenotypic plasticity. *L. dubia* [28] and *L. pectoralis* [31] could plastically develop long spines in the presence of fish predators. However, because of long-dispersing ability of adults [50], fish-lake species could also lay their eggs in invertebrate lakes, and vice versa. Therefore, local adaptation in response to the current predation habitat seems unlikely. Further, we raised all larvae without any predators’ cues; thus, plasticity within this generation could be excluded. The divergence of spine ontogenetic patterns most probably evolves as a long-term adaptation based on their preferred top predators. Moreover, a close inspection on the cross-generation coevolution of *Leucorrhinia* larvae and co-existing predators will be greatly helpful in understanding the evolution of ontogenetic patterns of defensive spines. However, although we could tell the presence of predatory fish by sampling in recent years, the detailed information on the historical changes of predator composition in the lakes could not be found. Therefore, much more effort needs to go into clearly unveiling the evolutionary mechanisms of ontogenetic pattern diversification in *Leucorrhinia* spines.

Overall, our results showed that different predators drive differentiation of ontogenetic growth patterns in an antipredator traits and growth of defensive spines is most pronounced during ontogeny when the potential benefits seem largest. The impacts from predators are most often obvious in the final morphological stage but studying ontogenetic patterns of antipredator traits clearly increases our understanding how selection shapes expression of antipredator traits.

## Figures and Tables

**Table 1:** AIC values of fitting models for lateral spines in segment 8 and 9 across *Leucorrhinia* species.

Species	*L. dubia*		*L. rubicunda*		*L. caudalis*		*L. albifrons*		*L. pectoralis*	
Spines	L9	L8	L9	L8	L9	L8	L9	L8	L9	L8
monomolecular	−650.8	−802.2	−359.7	−456.9	NA	−256.8	NA	−769.7	−641.1	−736.1
3-parameter logistic	−674.2	−824.6	−377.2	−480.0	−240.3	−283.3	−585.1	−799.6	−685.1	−774.4
4-parameter logistic	−672.3	−822.6	−375.1	−482.4	−238.4	−281.7	−584.0	−798.6	−683.9	−774.3
Gompertz	−673.1	−823.8	−375.5	−474.1	−239.4	−284.0	−577.0	−794.3	−679.6	−768.8
linear	−636.9	−792.8	−343.5	−437.8	−205.4	−256.3	−549.7	−767.3	−640.3	−725.5
linear without intercept	−627.3	−779.2	−339.5	−439.7	−54.7	−217.4	−369.6	−696.8	−550.0	−718.5
exponential	−627.3	−761.2	−312.8	−414.9	−153.1	−228.9	−445.1	−687.4	−568.6	−677.9

NA indicates that the model does not fit for the data. Values with lowest AIC are highlighted with a gray background. L8 and L9 represent the lateral spines on segment 8 and 9.

## Data Availability

The raw data supporting the conclusions of this article will be made available by the authors, without undue reservation.

## Supplementary Materials

The following supporting information can be downloaded at: https://www.mdpi.com/article/10.3390/insects14080712/s1, Table S1: Models used for fitting scaling relationship between spines (S) and body size (H for head width); Table S2: The head width on the inflexion points (HIP) of the curves and its SE for each spine in *Leucorrhinia* species.
