# Supplementary material for: Allometry of Defense: Predator Shift Alters Ontogenetic Growth Patterns in an Antipredator Trait
Source: Insects. 2023 Aug 17;14(8):712. doi: 10.3390/insects14080712 (PMC10456028; doi:10.3390/insects14080712)
Supplement: Supplementary file 1 [file insects-14-00712-s001.zip › insects-2528473-supplementary.pdf]

Supplementary materials

Table S1. Models used for fitting scaling relationship between spines (S) and body size (H for head width). Absolute growth rate (AGR) is the derivative of S with respect to H, i.e. *d*S/*d*H. Relative growth rate (RGR) can be expressed either as a function of body size (H), i.e. (*d*S/*d*H)/H. Inflection point is the point of body size at which AGR is maximized.

Shape	Name	Description
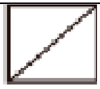	Linear	Constant AGR
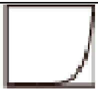	Exponential	Constant RGR
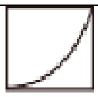	Power law	RGR will slow down with increasing of body size
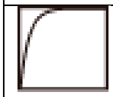	Monomolecular	AGR is fast initially and slows thereafter
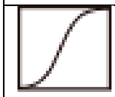	Three-parameter logistic	Asymptotic regression; lower horizontal asymptote is fixed at 0, the inflection point falls at K/2 (K is asymptotic spine length)
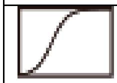	Four-parameter logistic	Loose one or the other of strictures in three-parameter logistic model
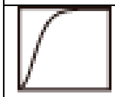	Gompertz	Inflection point occurs at around 37% of asymptotic spine length K

Reference: Paine et al. How to fit nonlinear plant growth models and calculate growth rates: an update for ecologists. *Methods in Ecology and Evolution*, 2012, 3, 245–256.

Table S2. The head width on the inflexion points (HIP) of the curves and its SE for each spine in *Leucorrhinia* species.

Species	HIP for lateral 9	SE for lateral 9	HIP for lateral 8	SE for lateral 8	Fitting model for spine 9	Fitting model for spine 8
L. albifrons	3.717	0.094	3.407	0.106	logis	logis
L. caudalis	4.199	0.122	3.501	0.124	logis	logis
L. dubia	2.902	0.055	2.970	0.061	logis	logis
L. pectoralis	3.632	0.070	3.426	0.088	logis	4pl
L. rubicunda	2.843	0.076	2.960	0.084	logis	4pl
